# Broader Autism Phenotype in Siblings of Children with ASD—A Review

**DOI:** 10.3390/ijms160613217

**Published:** 2015-06-10

**Authors:** Ewa Pisula, Karolina Ziegart-Sadowska

**Affiliations:** Faculty of Psychology, University of Warsaw, Stawki 5/7, 00-183 Warsaw, Poland; E-Mail: karolinaziegart@gmail.com

**Keywords:** broad autism phenotype, siblings, review, at-risk infants

## Abstract

Although less pronounced, social, cognitive, and personality characteristics associated with autism spectrum disorders (ASD) may be present in people who do not meet ASD diagnostic criteria, especially in first-degree relatives of individuals with ASD. Research on these characteristics, referred to as broader autism phenotype (BAP), provides valuable data on potential expressions of autism-specific deficits in the context of family relations. This paper offers a review of research on BAP in siblings of individuals with ASD, focusing on reports regarding social, communication, and cognitive deficits, published from 1993 to 2014. The studies are divided into two groups based on participants’ age: papers on preschool and older siblings of individuals with ASD; and publications on infants at risk for ASD. On the basis of this review, suggestions are offered for further research and its significance for our understanding of the genetic determinants of autism.

## 1. Introduction

Autism spectrum disorders (ASD) are neurodevelopmental disorders characterized by deficits in social communication and the presence of repetitive patterns of behavior, activity, and interests [1,2]. An important contributing factor in ASD etiology could be genetic liability, since the occurrence rate for ASD in siblings is on average 20- or 25-fold higher than in the general population [3,4,5].

The genetic mechanisms involved in the development of ASD are complex and heterogeneous [4,6,7]. This complexity is reflected by the variety of clinical characteristics of ASD, involving differences in the combination and expression of symptoms and severity of disorders in affected individuals. The heterogeneous, multifaceted nature of ASD is what drives research into the many possible expressions of autism that incorporate its typical deficits. The data obtained in that research may help us understand the genetic mechanisms that contribute to the development of autism-specific functional traits and atypical developmental trajectories. Moreover, this data may be useful in identifying genetic factors specific for various autism phenotypes, both in subjects with ASD and individuals from the general population.

A clear diagnostic conceptualization of autism still has yet to be established. It has been proposed that behavioral and cognitive characteristics of ASD, which include social, communication, and cognitive processes, rigid and persistent interests, and rigid and aloof personality traits, are continuously distributed throughout the general population [8]. These characteristics are likely to be more prevalent in first-degree relatives of individuals with ASD than in other groups [6,9,10,11,12,13,14,15]. Frazier and colleagues [16] studied how caregivers reported autism symptoms among children in their charge diagnosed with ASD and their unaffected siblings. They obtained data supporting the view that ASD is best characterized as a category. However, they note that “these data do not discount the possibility of endophenotypes or subthreshold ASD symptoms in unaffected family members” [16]. Wheelwright and colleagues [17] point out that studying ASD in nonclinical samples may be valuable in the context of the variety of genetic factors that seem to be connected to ASD. The genetic research on autism may benefit from a more inclusive concept of genetic expression that comprises cognitive, social, and communication deficits, as well as personality traits [18,19]. Characteristics similar to autism but less severe are referred to as broader autism phenotype (BAP) [20]. The notion of BAP facilitated involvement in studies on ASD and related phenomena of a large number of individuals without a clinical diagnosis of ASD. Consequently, it became possible to apply methods of the quantitative genetics typically used in the studies of normally distributed characteristics [21]. Research on BAP may help identify more functionally homogeneous subgroups of affected individuals and pinpoint the genetic factors that contribute to the development of ASD symptoms or traits [22]. The identification of phenotype may, therefore, open up the possibility of hypothetically assigning responsibility to a candidate gene or chromosome region. It therefore seems that data obtained in studies on BAP may provide promising cues for more detailed hypotheses on the genetic background of ASD. These studies may also contribute to a better understanding of the lesser variants of autism [23]. Since the data collected to date are not uniform, their applicability to genetic studies remains limited, and indicating the phenotypes with a clear genetic connection is still a difficult question. This may be due to the fact that the BAP concept covers a range of cognitive and social abilities and personality traits. Precisely establishing which characteristics should be included in BAP is a somewhat controversial undertaking. The lack of standardized criteria for BAP complicates attempts to engage in a comparative review of research on the topic. The features generally recognized as the most typical are deficits in social functioning, pragmatic language difficulties, restricted, repetitive behaviors and interests, as well as cognitive deficits (with respect to theory of mind in particular and social cognition in general, executive function, weak central coherence) and rigid personality [23]. Therefore, this paper focuses on the phenotypic characteristics of the individuals, observed from the perspective of clinical and experimental psychology. It seems that the concise summary of studies on BAP proposed here may provide some assistance in designing more advanced future studies aimed at identifying specific genetic mechanisms. Moreover, this paper will also demonstrate the methodological diversity in this field of study, which might help us in understanding the inconsistency of results across papers being published.

Gottesman and Gould [24] pointed out that phenotypes, understood as measurable characteristics, are heritable traits that are located in the path of pathogenesis from genetic predisposition to psychopathology. These traits are found at a higher rate in unaffected relatives of diagnosed family members than in the general population. The search for phenotypes may be conducted in specific populations that carry risk genes, but remain unaffected. Therefore unaffected relatives of individuals with the diagnosis, like healthy siblings, fulfill both criteria: they are enriched in risk genes and are healthy [25].

Therefore, a number of studies on BAP have been conducted on siblings of individuals with ASD. Initial findings have already indicated a significantly higher risk of autism in siblings than in the general population. Autism was initially reported to occur in approximately 2%–3% of siblings of affected children [26,27]. Folstein and Rutter [19], studying 21 pairs of same-sex siblings of whom one of the pairs was diagnosed with autism, found a concordance for autism in 4 of 11 sets of monozygotic twins, while in 10 dizygotic twin pairs they found no coexistence of autism in both children. Bolton and colleagues [28] analyzed family histories of autism in 137 individuals whose siblings had been diagnosed with the disorder, and found that 5.8% of siblings were diagnosed with autism, atypical autism, or Asperger syndrome, while no ASD diagnoses were reported among the siblings of individuals with Down syndrome. In a study by Ghaziuddin [29], 4.3% of siblings of 114 children with Asperger syndrome or autism were also diagnosed with Asperger syndrome or autism disorder.

Rates of BAP among siblings of individuals with ASD are much higher. August, Stewart, and Tsai [18] found cognitive disabilities in 15.5% of 71 siblings of probands with autism (compared to less than 3% in the control group). Folstein and Rutter [19] put forward a hypothesis that it is not autism as such that is inherited, but rather a pervasive cognitive deficit present both in individuals with autism and their immediate family. Currently, it is estimated that characteristics typical for ASD are present in at least 10%–20% of siblings of children diagnosed with ASD [28,30]. Ozonoff and colleagues [31], whose study involved a sample of 600 children in the USA and Canada, reported that autism-characteristic symptoms developed in nearly 19% of children whose older siblings had ASD. Boys are particularly at risk, with the correlation three times higher than in girls. At least one BAP trait is found in approximately 50% of family members of individuals with ASD when parents are included [32]. Biological siblings are attracting the attention of researchers studying BAP due to the large percentage of shared genes and environmental factors affecting their development.

Over the past few decades the corpus of data on BAP in siblings of individuals with ASD has expanded significantly. However, the studies in this area are highly diverse in terms of aspects such as design, instruments used, participant groups, as well as number of participants, their ages, and functions of interest, which lead to their findings being particularly difficult to integrate. Some help in overcoming that difficulty is offered by review papers that pool information on various aspects of studies and their results [23,33,34,35,36,37]. These reviews, especially those on preschool or older siblings, rarely provide information on participants’ age. Due to the neurodevelopmental nature of ASD and the specifics of the processes involved, the inclusion of that information would seem reasonable. In the light of increasing interest in studying infant siblings of children with ASD among researchers seeking early predictors of ASD, summarizing the data collected from siblings could provide valuable information. Moreover, the methods for collecting data from children, adolescents, and adults are different, which provides even greater encouragement to have a closer look at the studies on BAP in different groups of siblings of individuals with ASD. The present paper provides a review of research on BAP traits in siblings of individuals with ASD, wherein the body of research will be divided into two groups: preschool or older siblings, and at-risk infants. The two will be treated as distinct due to the different nature of studies on infant siblings of children with ASD compared to research on older siblings. The studies on infants and toddlers are quite often prospective, enrolling both healthy children and children later diagnosed with ASD. With repeated measures it is possible to follow the siblings’ developmental trajectories, unlike in most available studies on children over three or four years of age. Studies on at-risk infants have only emerged in the last decade; earlier research focused on older children, and as such these studies will be discussed first. For the reasons mentioned above, we have not attempted to propose a systematic review as defined in the PRISMA statement [38]. The present paper rather involves the subjective perspective of the authors reflected by the choice of the reviewed papers.

## 2. Research on Preschool-Age or Older Siblings

Table 1 presents a list of studies on broader autism phenotype in preschool or older siblings of individuals with ASD, published in the years 1993–2014. The list includes mainly studies that enrolled healthy siblings not affected by autism, although in a handful of them some participants were diagnosed with ASD or another disorder (*i.e.*, delayed language development) during the course of the study [28,39,40]. We have rejected studies in which the results of siblings were pooled in statistical analysis with the results of parents or other relatives *i.e.*, [41,42], which was sometimes a consequence of very small sibling subgroup size (like in [43]—only four siblings). Furthermore, publications included in the list meet the following criteria: (a) Peer-reviewed articles published in English; (b) Original studies; (c) Containing a control group; and (d) The social communication, language, and cognitive characteristics of autism in siblings of individuals with autism were the objects of study.

As shown in Table 1, studies that involve healthy siblings of individuals with ASD vary in design. They differ in terms of compared groups, sibling ages, sample sizes, analyzed aspects of functioning, as well as instruments and methods.

**Table 1:** Social communication, language, and cognitive BAP characteristics in siblings of individuals with ASD.

Study	Journal & Year	Characteristics	Sample	Age of Siblings of Individuals with ASD	Control Group, Matching Criteria	Measures/Instruments (Samples)	Main Findings
Ozonoff, Rogers, Farnham, & Pennington [45]	*J. Autism Dev. Disord.*, 1993	Executive function, theory of mind	18 Siblings of high functioning autism (HFA) children	8–18 years	18 siblings of children with learning disability, 18 HFA individuals, 18 learning-disabled children, matched on the basis of IQ, gender, socioeconomic status (SES), and ethnic background	Wechsler Intelligence Scale for Children–Revised (WISC-R), Wechsler Adults Intelligence Scale–Revised (WAIS-R), Wisconsin Card Sorting Test (WCST), Tower of Hanoi, Second-Order Belief Atribution Task, Fox and Grapes Task, Apple-Dog Task	HFA siblings performed worse than controls in the measure of planning (Tower of Hanoi). No differences between siblings groups in: set-shifting, working memory, and inhibitory control, theory-of-mind, Verbal IQ, Performance IQ, and Full Scale IQ
Szatmari *et al.* [45]	*J. Am. Acad. Child Adolesc. Psychiatry*, 1993	Cognitive impairments, adaptive behavior, developmental history	Siblings (and parents) of 52 pervasive developmental disorders (PDD) probands	6–18 years	Siblings (and parents) of 33 Down syndrome and low birth weight controls, matching criteria: IQ, family size, SES	Vineland Adaptive Behavior Scales (VABS), the Revised Stanford-Binet, Wide Range Achievement Test-Revised (WRAT-R), WCST	No differences between ASD siblings compared to control siblings on the social and communication domains of the VABS. No group differences in developmental history of language delays
Bolton *et al.* [28]	*J. Child Psychol. Psychiatry*, 1994	Social and communication impairments	137 Siblings (and 198 parents) of ASD individuals	Younger than 8 years	Down syndrome probands relatives (64 siblings, 72 parents); matching for age, sex, social class, birth order, and maternal age	Family History Interview, Autism Diagnostic Interview	20.4% of ASD siblings (and 3.1% of control siblings) demonstrated communication atypicalities, social impairments, or restricted behaviors; 4 out of 137 siblings met ICD-10 criteria for autism (two of them were mentally retarded), a further three were classified as having atypical autism and one was diagnosed with Asperger syndrome
Fombonne *et al.* [46]	*J. Child Psychol. Psychiatry*, 1997	General intellectual functioning, reading and spelling skills	Siblings (and parents) of 99 autism probands	Children and adults, lack of precise data	Siblings (and parents) of 36 Down syndrome individuals, matched for age, sex, social class, birth order, and maternal age with the autism probands	Family History Schedule, WAIS-R, WISC-R, Gray Oral Reading Test (GORT), Edinburgh Reading Tests, National Adult Reading Test, The Schonell Graded Word Spelling Test-B	Slightly higher mean verbal IQ scores in siblings of ASD individuals. Only the group of siblings of ASD individuals identified as affected with the BAP had significantly lower IQ scores and poorer reading and spelling abilities than unaffected siblings
Piven, Palmer, Jacobi, Childress, and Arndt [14]	*Am. J. Psychiatry*, 1997	Social and communication deficits	12 siblings from multiple incidence autism families	4–30 years	53 siblings of Down syndrome individuals; matched by probands age; no differences in parental education level and age	Family History Interview for Developmental Disorders of Cognition and Social Functioning	Higher rates of social deficits in siblings from families with multiple-incidence autism. No differences in the rates of communication deficits or stereotyped behaviors in siblings
Folstein *et al.* [47]	*J. Child Psychol. Psychiatry*, 1999	Pragmatic language, verbal IQ, reading and spelling skills	87 siblings (and 166 parents) of individuals with autism	5–46 years	64 siblings (and 75 parents) of individuals with Down syndrome; very similar age of siblings and parental education	WISC-R, WAIS-R, GORT, Kaufman Battery	No differences in verbal IQ scores, reading and spelling skills
Hughes, Hughes, Plumet, & Leboyer [48]	*J. Child Psychol. Psychiatry*, 1999	Executive function: verbal fluency, planning, flexibility	31 siblings of children with autism	5 years 8 months–19 years 11 months	32 siblings of children with developmental delay, 32 children from unaffected families (with no family history of ASD); similar family backgrounds (living in a low-income area)	Cambridge Neuropsychological Test Automated Battery, Multistage set-shifting task, akin to the WCST, Corsi Block Tapping task, Tower of London, Verbal Fluency task	Superior verbal and spatial span in siblings of children with autism; higher number of autism siblings than controls performed poorly on the verbal fluency tasks, planning, and set-shifting
Briskman, Happé, and Frith [49]	*J. Child Psychol. Psychiatry*, 2001	Everyday-life preferences and activities	19 siblings of children with autism	8–18 years	13 dyslexic siblings, 11 controls; matched on the basis of SES, parental education	Parental report	No differences between ASD siblings and other groups in everyday skills and preferences (parent-rated) with the exception of two boys subsequently diagnosed on the Autism Spectrum
Happé, Briskman and Frith [50]	*J. Child Psychol. Psychiatry*, 2001	Information processing, “Central coherence”	19 siblings of children with autism	8–18 years	14 dyslexia siblings, 17 controls; matched on the basis of SES, parental education; no difference in chronological age between autism group probands, dyslexia group probands, or normal controls	WISC-R, WISC-III, phonological measures (*i.e.*, the Reading tests); Experimental measures (*i.e.*, the Embedded Figures Test, Block Design Task, Titchener Circles Illusion Task)	Intact central coherence in siblings
Pilowsky, Yirmiya, Shalev, & Gross-Tsur [51]	*J. Child Psychol. Psychiatry*, 2003	Language abilities	27 siblings of children with autism	6 years–15 years 1 month	23 siblings of children with mental retardation of unknown etiology, 22 siblings of children with developmental language disorders; groups matched by siblings’ age, gender, birth order, family size, ethnicity, and family income and by probands’ gender and mental age	Children’s Evaluation of Language Fundamentals (CELF)	Higher scores in siblings of children with autism on receptive, expressive, and total language scales and on verbal IQ compared to siblings of children with developmental language disorders
Dorris, Espie, Knott, & Salt [52]	*J. Child Psychol. Psychiatry*, 2004	Mind-reading	27 siblings of children with Asperger syndrome (AS)	7 years 6 months–17 years	27 control children matched for age, sex, and a measure of verbal comprehension	“Eyes Test”, British Picture Vocabulary Scale II	Poorer performance of AS siblings in the “Eyes Test”
Bishop, Maybery, Wong, Maley, & Hallmayer [11]	*Am. J. Med. Genet. B: Neuropsychiatr. Genet.*, 2006	Communication deficits	43 ASD siblings	4–16 years	46 control children; matching criteria: age, sex	Parent’ report on Children’s Communication Checklist-2 (CCC-2)	The only difference between groups was syntax; 23.8% of ASD siblings scored 2 SD below the control mean on CCC-2, compared to 2.2% of controls. Some differences in structural language skills
Constantino *et al.* [6]	*Am. J. Psychiatry*, 2006	Subsyndromal autistic impairments	49 siblings of children with autism from multiple-incidence families, 100 siblings of children with any PDD	4–18 years	45 siblings of children with psychopathology unrelated to autism, no matching criteria discussed	Social Responsiveness Scale (SRS)	Siblings of children with autism from multiple-incidence families—the highest scores in the SRS; followed by siblings of probands with any PDD, and then siblings of the probands with psychopathology unrelated to autism
Shaked, Gamliel, & Yirmiya [53]	*Autism*, 2006	Theory of mind	24 siblings of children with ASD (SIBS-A)	54–57 months	24 typically developing siblings (SIBS-TD), matched by siblings’ age, gender, birth order, parents’ age, and education	The false belief and the strange stories tasks	No differences on both theory of mind tasks
Christ, Holt, White, & Green [54]	*J. Autism Dev. Disord.*, 2007	Executive function: inhibitory control, processing speed	21 siblings of children with ASD	6–15 years	18 children with ASD, 25 typically developing (TD) controls, matched on age, overall IQ, and processing speed	Stroop Card Task, Stroop Computer Task, Flanker Task, Go/No-go Task	No differences between ASD siblings and controls in processing speed and inhibitory control
Chuthapisith, Ruangdaraganon, Sombuntham, & Roongpraiwan [39]	*Autism*, 2007	Language development	32 preschool siblings of children with autism	2–6 years	28 control children, matched by siblings’ age, gender, maternal educational level and family income	The Stanford-Binet IV	Delayed language development in eight of the autism siblings. Following exclusion of siblings with ASD and developmental language disorder (DLD) diagnosis, the remaining 29 siblings’ verbal IQs were not significantly different from the control group
Dalton, Nacewicz, Alexander, & Davidson [55]	*Biol. Psychiatry*, 2007	Face processing	12 ASD siblings	8–18 years	21 individuals with autism, 19 TD controls, matched for age and intelligence quotient (IQ)	Facial Recognition Task, eye tracking, brain functional magnetic resonance imaging	Decreased gaze fixation and brain function in response to images of human faces in ASD siblings; less time than the control group fixating the eye region in response to naturalistic photographs of both familiar and unfamiliar human faces
Gamliel, Yirmiya, & Sigman [56]	*J. Autism Dev. Disord.*, 2007	Cognitive and language development—a prospective study	39 ASD siblings (SIBS-A)	4–54 months	39 siblings of TD children (SIBS-TD); matched at 4 months according to chronological age, sex, birth order, number of children in the family, sex of the older proband, temperament profile, and mental and motor scores	Bayley Scales of Infant Development—2nd Edition (BSID-II), Reynell Developmental Language Scales (RDLS), Kaufman Assessment Battery for Children (K-ABC), Clinical Evaluation of Language Fundamentals-Preschool (CELF-P)	A delay in cognition and/or language in 12 of the SIBS-A and only two SIBS-TD; one child subsequently diagnosed with autism. Cognitive differences disappeared by age 54 months, while some differences in receptive and expressive language abilities remained. Most SIBS-A were well-functioning
Pilowsky, Yirmiya, Gross-Tsur, & Shalev [40]	*J. Autism Dev. Disord.*, 2007	Neurocognitive functioning (*i.e.*, intellectual abilities, acquired knowledge and achievement, executive function, attention and distractibility, sequential and simultaneous processing), behavior problems, developmental history, language abilities	30 siblings of children with autism	6–16 years	28 siblings of children with mental retardation, 30 siblings of children with developmental language delay; matched by siblings’ chronological age, gender, birth order, probands’ gender, and family income	WISC-III, WRAT-III, Tower of Hanoi, CELF-III (Word Associations Test), Rapid Automatic Naming test, Attention Deficit and Hyperactivity Questionnaire, Sequences Test, Visual Perception Test, Child Behavior Checklist, Family History Questionnaire	After excluding from ASD sibling group two siblings diagnosed with PDD, there were no differences between siblings of children with autism and the other groups
Gamliel, Yirmiya, Jaffe, Manor, & Sigman [57]	*J. Autism Dev. Disord.*, 2009	Cognitive and language development–a prospective study	37 siblings of children with ASD (SIBS-A)	4 months–7 years	47 siblings of TD children (SIBS-TD); matched at 4 months on the basis of age, sex, birth order, number of children in the family, sex of the older proband, and temperament profile	BSID-II, K-ABC, RDLS, CELF-P, WISC-III, WRAT-III, CELF-III	At 7 years, 40% of the SIBS-A (and 16% of SIBS-TD) showed cognitive, language and/or academic difficulties (this sub-group was named SIBS-A-BP). Early language scores (14–54 months) were significantly lower in SIBS-A-BP compared to the language scores of SIBS-TD. Language as a major area of difficulty for SIBS-A during the preschool years
Kawakubo *et al.* [58]	*PLoS ONE*, 2009	Verbal fluency	24 siblings of children with ASD	Children (*n* = 12; M age = 11.1; SD = 3); Adults (*n* = 12)	27 high functioning individuals with ASD, 27 unrelated healthy controls with no family history of ASD, matched for age and IQ	Letter fluency task	No differences between either the child or the adult group
Koh, Milne, & Dobkins [59]	*Neuropsychologia*, 2010	Motor perception	13 adolescents with siblings diagnosed with ASD	13 years–17 years 11 months	23 adolescents with ASD, 42 TD adolescents, matched for age and gender	Two experiments: “detection task”, and the “motion task”	SIBS showed higher chromatic contrast sensitivity than both participants with ASD and TD participants, what authors interpreted as the possible existence of a protective factor in these individuals against developing ASD
Ben-Yizhak *et al.* [60]	*J. Autism Dev. Disord.*, 2011	Pragmatic language, school related linguistic abilities	35 siblings of children with autism (SIBS-A)	9–12 years	42 siblings of TD children; matching criteria at age 4 months: chronological age, sex, birth order, number of children in the family, sex of the older proband, temperament profile, and mental and motor scores	ADOS, SCQ, WISC-III, CELF-III, WRAT-III) Diagnostic Battery for Reading Processes in Hebrew	Lower pragmatic language abilities in a subgroup of SIBS-A identified with BAP-related difficulties. No differences between groups in general linguistic measures, school achievements, and reading processes
Levy and Bar-Yuda [61]	*Autism*, 2011	Language performance	28 siblings of nonverbal children with autism SIBS-ANV	4–9 years	27 controls matched for age, family background, socioeconomic status, and type of school they attended	CELF, spontaneous speech samples	SIBS-ANV achieved lower scores on the Receptive Scale, Expressive Scale and the Total Language Scale of the CELF; differences in the language scores were associated with IQ. No differences between groups in the results of grammatical analysis of spontaneous speech samples
Sumiyoshi, Kawakubo, Suga, Sumiyoshi, & Kasai [62]	*Neurosci. Res.*, 2011	Ability to organize information, executive function	14 siblings of individuals with ASD	Adults: M age = 24.5; SD = 4.0	22 individuals with ASD, 15 age-matched control subjects	WCST, Verbal Learning Task (VLT), AQ, CARS	No differences between siblings and controls in the WCST and VLT results. Authors noticed that a linear increase of the memory organization score in the VLT was absent in siblings as well as the ASD group. More autistic traits measured by AQ and CARS in siblings than in controls
Fiorentini *et al.* [63]	*Neuropsychologia*, 2012	Face identity	8 siblings (and 20 parents) of ASD children	7 years 11 months–16 years 3 months	10 TD children, (and 20 parents of TD children); matched for age, IQ	Face identity after effect task	Face-coding mechanisms in relatives of ASD individuals similar but less efficient compared to the relatives of typical children
Warren *et al.* [64]	*J. Autism Dev. Disord.*, 2012	Neurocognitive, language, and behavior measures—a prospective study	39 younger siblings of children with ASD (SIBS-ASD)	4 years 2 months–7 years 4 months	22 younger siblings of TD children; matching criteria: gender, chronological age, and SES	Differential Ability Scales—Second Edition, NEPSY-II, CELF-P, Children’s Communication Checklist-2 for Parents, ADOS, SRS, CBCL, Social Skills Rating System	Executive functioning composite, Auditory attention, Inhibition–naming–worse in SIBS-ASD compared to controls. No differences in CBCL results
Gerdts, Bernier, Dawson, & Estes [65]	*J. Autism Dev. Disord.*, 2013	Broader autism phenotype traits as measured by Broader Phenotype Autism Symptom Scale	Siblings and parents from 87 multiplex and 41 simplex ASD families	Simplex families: M age = 11.51 (SD = 3.59); Multiplex families: M age = 10.20 (SD = 4.20)	Only members of ASD families	Broader Phenotype Autism Symptom Scale	Siblings from multiplex ASD families revealed less social interest, poorer conversational skills, higher rigidity, and intense interests and were less expressive in the use of nonverbal communication than siblings from simplex ASD families
Malesa *et al.* [66]	*Autism*, 2013	Prospective study–follow-up evaluation at age 5 years	38 from 54 later-born SIBS-ASD, participating in the original study by Yoder *et al.* [67] (see: Table 2)	4–7 years	23 from 31 later-born SIBS-TD, participating in the original study; matching criteria: chronological age, race, and gender	ADOS, ADI-R, CELF-P, CELF-4, Differential Ability Scales–Second Edition (DAS-II), Social Skills Rating System (SSRS)	Two SIBS-ASD received a diagnosis of ASD at follow-up; none of the SIBS-TD received a clinical diagnosis. At age five there were no differences between SIBS-ASD and SIBS-TD in performance on most social and language domains assessed with standardized measures
Oerlemans *et al.* [68]	*J. Autism Dev. Disord.*, 2013	Executive function, social cognition, local processing style	172 siblings of children with ASD	6–21 years	140 Children with ASD, 127 controls; matched on the basis of age and ethnic background	Face recognition task, the Identification of Facial Emotions task, the Prosody task, Go/No-Go task, The Response Organization Objects task, Digit Span task (from Wechsler Scale)	ASD siblings performed worse than controls in face recognition task and inhibition task (but the differences referred only to processing speed)
Pickles, St Clair, & Conti-Ramsden [69]	*J. Autism Dev. Disord.*, 2013	Communication and social deficits	134 siblings (and 193 parents) of probands with ASD	8–42 years	Specific Language Impairment (SLI-only, 79 siblings, 103 parents), SLI + ASD (43 siblings, 30 parents), Down syndrome (DS, 63 siblings, 70 parents), matched on the basis of age and other criteria (not listed precisely)	Family History Information (with modifications)	ASD and SLI siblings had higher levels of communication deficits in relation to DS siblings (especially in the rate of language delay, level of spelling difficulties). More social deficits in ASD relatives in comparison to DS and SLI-only relatives
Robel *et al.* [70]	*Eur. Child Adolesc. Psychiatry*, 2013	Autistic traits	24 siblings of children with autism	M age = 9.4 (SD = 1.82)	96 siblings of TD children, aged three or older, from different socioeconomic backgrounds	French Autism Quotient (two main factors: F1 corresponding to socialization and communication, F2 to imagination and rigidity)	F1 and total AQ score higher in siblings of children with autism and global scores; no differences in F2 scores
Gizzonio *et al.* [71]	*Exp. Brain Res.*, 2014	Cognitive profile	21 siblings of children with ASD	6–16 years	32 Affected with ASD brothers of participating siblings, 43 TD children; matching criteria not listed precisely	WISC-III, SRS	No significant differences between Verbal Intelligence Quotient and Performance Intelligent Quotient scores among groups. Not significant, predominance of performance over verbal abilities observed in siblings group. Common cognitive profile in ASD group and ASD siblings
Holt *et al.* [22]	*Psychol. Med.*, 2014	Theory of mind	40 full siblings of individuals with autism or AS	12–18 years	50 adolescents with HFA or AS, 40 TD controls; matching criteria: age, full scale IQ above 70	“Reading the Mind in the Eyes” task	No differences between siblings and controls
Oerlemans *et al.* [72]	*Eur. Child Adolesc. Psychiatry*, 2014	Recognition of facial emotion and affective prosody; verbal attention	79 siblings of children with ASD	6–13 years	90 children with ASD (43 with and 47 without ADHD), 139 controls; matched on the basis of age and ethnic background	Facial emotion and affective prosody experimental tasks	Poorer performance of unaffected siblings than controls and better than the ASD probands in recognition of facial emotion and affective prosody tasks

### 2.1. Comparison Groups

All papers listed in Table 1 included one or more comparison groups. One exception is the paper by Gerdts *et al.* [65], which is listed despite the fact that only ASD families were included in that study. However, the inclusion of multiplex and simplex ASD families provides valuable insight into the severity of BAP traits in families with more than one child with autism.

Analysis of the groups used in comparisons with siblings of individuals with ASD reveals several strategies adopted by researchers. The most commonly-used comparison group consists of healthy siblings of typically developing individuals, or simply typically developing individuals themselves with no family history of autism [48,54,60,63,64,70]. This strategy allows for a comparison of the development of ASD siblings with their typically developing peers from families with no autism.

In many studies, one of the comparison groups was composed of individuals diagnosed with ASD, usually high-functioning autism or Asperger syndrome [22,44,54,55,58,59,62,68,72]. This way, the results of siblings could be compared with those obtained from individuals with ASD, and it was possible to determine whether the instruments used actually identified these deficits. In some of these studies, the results achieved by siblings of individuals with ASD landed in the middle, between the scores of individuals with ASD and those achieved by typically developing controls *i.e.*, [62,71]. This could indicate qualitatively similar traits or behavioral profiles of siblings and individuals with ASD, although the results of siblings are closer to the developmental norm. Some studies evaluated affected and unaffected siblings from the same family, *i.e.*, [71]. This empirical approach has particular merit in light of the fact that siblings share on average 50% of genes with their affected brother or sister [68]. Verifying similarities between siblings in terms of autistic traits may offer new insights into genetic susceptibility to autism.

In several studies, typically older ones, the comparison group for siblings of individuals with ASD was composed of siblings of individuals with Down syndrome, *i.e.*, [14,45,46,47,69]. This was to control for the effects of a family member with a hereditary disability not associated with ASD on the development and functioning of siblings. Recently, however, the focus has been on the comorbidity of Autism Spectrum Disorders and Down syndrome. Lowenthal *et al.* [73] showed that the frequency of Pervasive Developmental Disorders in individuals with Down syndrome was 15.6%, including 5.58% for autism. These findings must be taken into account in the selection of siblings of individuals with Down syndrome for the group compared with the siblings of ASD individuals. The presence and severity of autistic traits and symptoms in probands with Down syndrome must also be controlled for. With the high incidence of ASD (see [74]), this should be a universally-observed principle applied in the selection of comparison groups in studies on BAP, not limited to Down syndrome participants.

In some research projects, comparison groups consisted of siblings of individuals with developmental delay [48], mental retardation of unknown etiology [40,51], or psychopathology unrelated to autism, such as ADHD, affective disorders, and anxiety disorders [6]. There is also an interesting group of studies that included siblings of children with such developmental problems as dyslexia [49,50] and specific language impairment [69]. Extensive research has recently been done on potential associations between these complex developmental disorders and autism as, similarly to autism, their incidence is greater in males, they involve a number of neurophysiological and neuroanatomical abnormalities, they are likely to have strong genetic components, and they encompass language and communication deficits [75,76].

There is no question that the type of comparison groups should be taken into consideration when interpreting results and forming generalizations regarding the presence or absence of specificity in the functioning of siblings of individuals with ASD. In the case of heritable conditions that share features with ASD (e.g., SLI), it is possible that the first-degree relatives of probands will present some characteristics that overlap with the BAP [23]. This may cause additional difficulties in comparisons and the isolation of the autism phenotype.

### 2.2. Participants’ Age and Sample Size

In the majority of studies listed in Table 1, the participants’ functioning was measured only once. The exceptions are two longitudinal studies by Gamliel and colleagues [56,57] that include children aged 4 months to 4.6 and 7 years, and studies by Warren *et al.* [64] and Malesa *et al.* [66], which were a continuation of a study by Yoder *et al.* [67]. As these studies provide information about preschool and school-age children, we decided to include them in Table 1. There is only one other study [39] in which participants were under 4 years of age. In all other works the siblings of individuals with ASD were aged 4 or older, and in a number of them the age range was quite large. In some papers it exceeded 10 years, *i.e.*, [44,49,55], and could be as high as 40 years, *i.e.*, [47,69].

The age of siblings at the time of the study is not always crucial; in designs where caregivers were asked to provide information on the child’s development history, that variable is less significant *i.e.*, [46,69]. However, in those studies that measure developmental levels across various domains or mastery of specific functions, a wide age range of participants may compromise the precision of inferences. It also cannot capture any developmental delays or irregularities that may be present during a certain period. However, knowledge of the specifics of that aspect of development could help in planning support for children in ASD families. A wide sibling age range makes it difficult to determine the level of that group’s functioning, especially when coupled with a small sample size, which precludes statistical analysis on subgroups more homogenous in terms of age. In some studies the ASD siblings group counted under 20 participants *i.e.*, [14,44,49,50,55,59,62,63]. There is, however, a clear trend towards increased sample size in more recent studies, *i.e.*, [68,69]. In turn, small groups tend to be more homogenous, including in respect of participants’ age. One illustration is the study by Warren *et al.* [64], which enrolled 40 siblings aged 4 to 7; another comes from research on preschool and early school children aged 2 to 9 years *i.e.*, [39,61]. Ben-Yizhak *et al.* [60] assessed older children in a narrow age range from 9 to 12 years, while Koh *et al.* [59] and Holt *et al.* [22] evaluated adolescents aged 12–18. Nevertheless, the majority of studies are conducted on children and adolescents with wider age ranges: from 6–8 to 16–18 years, *i.e.*, [6,40,54,71].

As we have previously mentioned, research on siblings who are of preschool age, or on older children and adolescents, suffers from a lack of longitudinal studies. In one of a handful of reports that provide such information, Gamliel *et al.* [56] stated that cognitive deficits originally present in siblings of children with ASD disappeared during the preschool period (age: 54 months), but—compared to controls—some differences in terms of receptive and expressive language abilities remained. The results indicating differences in language development were confirmed in the next phase of the study, conducted when the children reached 7 years of age. Data of that sort is particularly relevant for understanding the specifics of developmental processes in children from ASD-affected families. While not meeting the diagnostic criteria for the disorder, these children may experience difficulties due to atypical development. Some of these difficulties are resolved, but there are some that may compromise their adjustment at a given moment in life. Thus, longitudinal studies that enable us to follow developmental processes, as well as to identify their determinants and effective methods of developmental support, are particularly valuable.

### 2.3. Functioning Characteristics of Interest

The studies listed in Table 1 cover a wide variety of characteristics. In some, non-affected siblings were assessed for autism symptomatology with instruments used in diagnosis (Autism Diagnostic Observation Schedule, ADOS [77] or Autism Diagnostic Interview-Revised, ADI-R [78]) or based on developmental history established from medical history or interviews other than ADI-R *i.e.*, [14,28,69]. The results of these studies suggest a greater incidence of deficits in at least one domain typical for ASD. However, their results are not fully consistent in terms of the domains affected by the deficits and the depth of the deficits in question.

Greater severity of autistic traits in the siblings of individuals with ASD compared to controls was shown in those studies that measured such traits using the Social Communication Questionnaire (SCQ [79]), Childhood Autism Rating Scale (CARS [80]), Autism Spectrum Quotient (AQ [8]) and Broader Phenotype Autism Symptom Scale (BPASS [32]) [6,62,65,79]. Another finding was that autistic traits, including less social interest, poorer conversational skills, higher rigidity, and intense interests, as well as less expressiveness in the use of nonverbal communication, are more pronounced in siblings from multiplex ASD families than in children from simplex ASD families [65]. Based on similar results obtained for social and communication domains of BAP in parents, Bernier *et al.* [81] concluded that different genetic transfer mechanisms may operate in families with one and families with several children with autism displaying many of these characteristics. It has been suggested that *de novo* mutations and non-inherited copy number variants may be particularly important risk factors in simplex ASD families, while in multiplex ASD families they are present to a much lesser degree [82]. However, this finding was not confirmed in all studies [83]. As noted by Spiker and colleagues [84], the variability of autistic phenotype expressed in multiplex families is relatively low. In their study on families with two or more children with autism, in 37 out of 44 participating families at least two children met the ADI diagnostic criteria. Out of all the children in these families, 71% met all of the criteria for autism diagnosis in ADI, 22% failed to meet any of the criteria, and 7% met one or more criteria without reaching all of the ADI cutpoints. The number of items classified as “uncertain” was small, and there was a clear-cut distinction between children affected and unaffected by autism. It should also be noted that studies involving siblings from simplex ASD families, siblings from multiplex ASD families, and controls are scarce. This reduces the possibility of identifying the scope of the differences between the three groups with respect to the BAP.

A substantial body of research into the functioning of ASD siblings concerns language and communication as part of the phenotype(s) of ASD. Findings vary, as do methods and instruments. In their grammatical analysis of spontaneous speech samples, Levy and Bar-Yuda [61] found no significant differences between ASD siblings and preschool controls. In some studies ASD siblings were found to be more likely to have language delays relative to their comparison groups [39,56,57], while in other papers such differences were not stated, *i.e.*, [45]. A frequently encountered pattern of results is one in which the group of ASD siblings as a whole does not differ from controls, but a subgroup may be distinguished that demonstrates more pronounced BAP traits, including inferior language development, *i.e.*, [39,60]. Interesting data comes from comparisons of ASD siblings with siblings of individuals with language disorders. Some researchers found less severe difficulties in language and communication in siblings of individuals with ASD than siblings of people with language disorders, *i.e.*, [51], while others demonstrated that ASD siblings, similar to specific language impairment siblings, demonstrate higher levels of communication deficits in relation to siblings of individuals with Down syndrome [69].

A number of studies analyzed siblings’ intellectual abilities [44,46,47,51,71]. This is an important issue since intellectual disability (ID) and autism are highly co-morbid [85]. It is estimated that ID is present in 50%–70% of all ASD cases [85]. Precise estimation of the co-morbidity of ASD and ID is complicated due to changing criteria, diagnostic procedures, and educational policy (e.g., providing more support to certain groups of pupils) as well as the methodological problems (screening tools properties) [86]. Nevertheless, it has been shown that the number of high-functioning individuals with ASD diagnosis has increased in recent years (see [74]). It is also noteworthy that IQ may be underestimated in people with high levels of autistic traits [87]. However, the interrelationships among the level of autistic traits and intellectual abilities are still far from being fully recognized, and the results of studies on IQ in siblings of individuals with ASD are equally difficult to generalize. In some of them, no differences were found between ASD siblings and controls in terms of IQ levels (e.g., [40,44,47]), while Fombonne *et al.* [46] and Pilowsky *et al.* [51] reported even higher verbal IQ in ASD siblings. Gizzonio *et al.* [71] found no differences between ASD siblings and controls in terms of Verbal IQ and Performance IQ, but reported a slight (non-significant) predominance of performance over verbal abilities. As the authors have suggested, this could indicate the presence of a certain cognitive profile common for individuals with ASD and their siblings, but statistically non-significant differences make this a very tentative conclusion. Fombonne *et al.* [46] identified a subgroup of participants who demonstrated BAP traits (referred to as BAP+) and had significantly lower IQ scores than the group of siblings non-affected with BAP. Similar results were reported by Chuthapisith *et al.* [39].

There have been numerous studies on autism-specific cognitive deficits: in theory of mind, central coherence, and executive function. Research on theory of mind, recognition of facial emotions, and face processing indicate that they are less developed in siblings of individuals with ASD compared to controls, *i.e.*, [55,63,68,72]. By contrast, the results of a study using the “Reading the Mind in the Eyes” task were inconclusive. Dorris *et al.* [52] showed that siblings scored lower than controls, but Holt *et al.* [22] found no differences between adolescent ASD siblings and typically developing control adolescents. Similarly, no differences in theory of mind between siblings and controls were reported by Ozonoff [44]. The key in studies of this type is to take into account the age of participating siblings, which is often overlooked. It is also noteworthy that this area of study is still strongly affected by a lack of precise conceptual definitions and methodological scrutiny, which makes the discussion even more complex. It must be noted that the results of studies on deficits discussed above in individuals with ASD are mixed, and are far from conclusive. A detailed discussion of the definitional controversies relating to particular aspects of cognitive deficits typical for ASD, as well as a presentation of current opinion concerning theory of mind, emotion recognition, central coherence, and executive function in people with ASD, remain outside the scope of this paper.

A complex picture also emerges from research on executive function. Siblings of individuals with ASD were found to be no different from controls in terms of inhibitory control and processing speed [54], but performed worse in planning tasks [44,48]. Ozonoff and colleagues [44] found no differences in working memory and set-shifting, while Hughes *et al.* [48] concluded that a larger than expected proportion of siblings of individuals with autism demonstrated difficulties in set-shifting. In the study conducted by Pilowsky *et al.* [40], differences in executive function between ASD siblings and controls disappeared once two participants diagnosed with Pervasive Developmental Disorders were removed from the former group. It should be also mentioned that Happe, Briskman, and Frith [50] reported no differences in terms of weak central coherence. In recent years the field of research on siblings of individuals diagnosed with ASD has been dominated by studies focused on infants at high familial risk for ASD (see [35]). It is estimated that about 10%–20% of high-risk infants can be affected with subclinical ASD traits or other developmental problems [88]. It should be emphasized that the analyses in some of the studies on infants also included children who later received an ASD diagnosis, which is why these results should be approached with caution. Nevertheless, research on infant siblings of children with ASD can not only provide us with valuable information on early signs of autism, but also pave the way for investigation of BAP traits [35].

## 3. Research on High-Risk Infants

Most studies focus on infant siblings of older children diagnosed with ASD, aged from 4 to 24 or 36 months. Diagnosis of the ASD proband is usually confirmed with ADOS and ADI-R outcomes, and the age of the proband is not relevant. Infant siblings are usually participants in long-lasting longitudinal studies in which different aspects of infants’ functioning are assessed, *i.e.*, [56,77,89]. The control groups mainly consist of typically developing infants without familial risk for ASD. Some research projects perform comparison analyses between high-risk infants (HR, siblings of older children diagnosed with ASD) and low-risk infants (LR, infants without familial risk for ASD) [90,91,92,93,94], while others strive for interpretation of infants’ functioning and test performance in the context of later ASD diagnoses or BAP characteristics [30,95,96,97,98]. Many studies differ in the sizes of HR and LR infant samples involved in analysis (*i.e.*, nine HR infants in [99] to 507 HR infants in [88]), making interpretation and comparison of results problematic.

A number of research projects on infant siblings are focused on early characteristics of ASD core symptoms, as well as overall risk for developing ASD, *i.e.*, [88,94,100,101,102]. Macari *et al.* [101] suggest that two thirds of infants at high risk for ASD experience some kind of developmental difficulties in the second year of life. A large study conducted by Messinger *et al.* [88] indicated elevated levels of autistic traits (higher mean ADOS severity scores) in HR infants compared to LR infants. Georgiades *et al.* [100] suggested that significantly more HR infants had exhibited higher levels of autistic-like traits than LR children, and at the age of three years these children had more social communication impairments, lower cognitive abilities, and more internalizing problems than typically developing children.

As shown in Table 2, many authors report early communication and language deficits [56,89,90,94,96,102,103,104], social interaction impairments [91,94,97,98,105,106,107], and increased levels of stereotyped behaviors [108,109,110] in HR infant siblings.

It should be noted that the emergence of some of the highlighted differences are probably caused by inclusion in analyses of high-risk infant siblings who later developed ASD. For instance, Rozga *et al.* [97] ascertained that high-risk infant siblings who later developed ASD exhibited lower rates of joint attention and requesting behavior than typically developing children. However, HR infant siblings without ASD outcomes did not differ in these characteristics from the control infants. Similarly, Bedford *et al.* [111] indicated that only the high-risk infants with later emerging socio-communication difficulties (ASD and atypical development) differed significantly from the control group in the gaze following task. What is more, high-risk infants without ASD outcome performed similarly to typically developing infants. Hutman *et al.* [95] also suggest social impairments only in the high-risk infants with later ASD diagnosis. Hudry *et al.* [104] observed reduced receptive vocabulary advantage in all HR infants by 14 months, but this difference was maintained through 24 months only in children with ASD outcome, while typically developing HR infants regained a more normative profile.

Some empirical data suggest the presence of deficits in quality of mother–infant interaction and differences in responses to separation events in high-risk infant siblings. For instance, Esposito *et al.* [112] found differences in cry sample patterns in HR toddlers compared to LR toddlers in expression of distress during the separation phase. However, Haltigan *et al.* [113] concluded that HR infants are not less likely to form secure affectional bonds with their caregivers than LR infants, but also mentioned that infant siblings of children with ASD are less distressed during separation and more reserved after reunion with a caregiver compared to typically developing children. Finally, a study conducted by Wan *et al.* [114] indicated that infant attentiveness to parent and positive affect were lower in the high-risk group later diagnosed with ASD. These characteristics, as well as dyadic mutuality, predicted a three-year ASD outcome.

A number of studies analyzed face processing and social visual fixation patterns as predictors of autistic traits in infants. Some HR siblings demonstrated diminished gaze to the mother’s eyes relative to her mouth in the Still Face episode [93]. During the face processing task, LR infants demonstrated a preference for looking at the left side of the face (characteristic left visual field bias) that emerged by 11 months of age and was absent in HR infants at any age [115]. Hutman *et al.* [95] observed no difference in the proportion of attention to social stimuli or attention shifting during the play condition between HR and LR infants. However, children later diagnosed with ASD tended to continue looking at a toy during the distress condition despite the salience of social information. Overall, no group differences between HR and LR infants in gaze following behavior at either age was observed in the study by Bedford *et al.* [111]. Nevertheless, it should be noted that HR infants with later emerging socio-communication difficulties (ASD and atypical development) allocated less attention to a congruent object compared to typically developing HR siblings and LR controls.

**Table 2:** Studies on infants and toddlers at risk for ASD.

Study	Journal &Year	Characteristics	Sample	Age of Siblings of Individuals with ASD	Control Group, Matching Criteria	Measures/Instruments (Samples)	Main Findings
Goldberg *et al.* [105]	*J. Autism Dev. Disord.*, 2005	Social communication behaviors	8 children diagnosed with ASD; 8 younger siblings of children with ASD	Below 3 years old	9 TD children, age and IQ controlled	ADI-R, ADOS-G, CARS, Early Social Communication Scales (ESCS)	On three of four of the ESCS subscales (Responds to Social Interaction, Initiates of Joint Attention, and Requesting Behaviors) social communicative behaviors of younger siblings differed from those of typically developing children but not from the behaviors displayed by ASD group
Zwaigenbaum *et al.* [102]	*Int. J. Dev. Neurosci.*, 2005	Autistic traits, autism-specific behavior	65 siblings of children with ASD	6 to 24 months	75 low risk infants, gender-, birth-order, and age-matched to high-risk infants	Novel observational scale a computerized visual orienting task, and standardized measures of temperament, cognitive and language development	Lower receptive language scores and use of fewer gestures and phrases at 24 months in non-autistic siblings. By 12 months of age, siblings who are later diagnosed with autism may be distinguished from other siblings and low-risk controls on the basis of: (1) behavioral markers, including atypicalities in eye contact or visual tracking; (2) prolonged latency to disengage visual attention; (3) a characteristic pattern of early temperament, with marked passivity and decreased activity level at six months; and (4) delayed expressive and receptive language
Landa and Garrett-Mayer [30]	*J. Child Psychol. Psychiatry*, 2006	Autistic traits, autism-specific behavior	60 HR infants (siblings of children with autism, SIBS-A) and 27 LR infants (no family history of autism), at 24 months of age categorized as: unaffected, ASD, or language delayed (LD)	6–24 months	27 low risk infants (no family history of autism), age, ethnic group, and SES were controlled	Language test scores, ADOS, MSEL	Lower scores on all MSEL scales in SIB-A at 14 months, compared to LD and TD children. By 14 months of age, the ASD group performed significantly worse than the unaffected group on all scales except Visual Reception. By 24 months of age, the ASD group performed significantly worse than the unaffected group in all domains, and worse than the language delayed group in Gross Motor, Fine Motor, and Receptive Language
Mitchell *et al.* [96]	*J. Dev. Behav. Pediatr.*, 2006	Early language and communication development	97 SIBS-A (then part of them diagnosed with ASD)	12 to 24 months	49 control children, recruited from three regions in numbers roughly proportionate to each region’s high-risk siblings	MacArthur Communicative Development Inventory (CDI), Preschool Language Scale—Third Edition, MSEL	Children with ASD showed delays in early language and communication compared with non-ASD siblings and controls. At 12 months, the ASD group was reported to understand significantly fewer phrases and to produce fewer gestures. At 18 months, they showed delays in their understanding of phrases, comprehension and production of single words, and use of gestures. Siblings not diagnosed with ASD also used fewer play-related gestures at 18 months than low-risk controls, even when children with identified language delays were excluded
Yirmiya *et al.* [90]	*J. Child Psychol. Psychiatry*, 2006	Social engagement, communication, and cognition	21 SIBS-A	4–14 months	21 TD infants, age-matched	Bayley Scales of Infant Development–2nd edition, Infant Characteristics Questionnaire (ICQ), Still-face paradigm, Name-calling responsiveness, Early Social Communication Scales (ESCS), Checklist for Autism in Toddlers (CHAT)	At 14 months, SIBS-A made fewer nonverbal requesting gestures and achieved lower language scores on the Bayley Scale. Infant SIBS-A, who showed more neutral affect to the still face and were less able to respond to their name being called by their mothers, initiated fewer nonverbal joint attention and requesting behaviors at 14 months, respectively
Bryson *et al.* [99]	*J. Autism Dev. Disord.*, 2007	Autistic traits, IQ	9 HR infants with older siblings with ASD, all of them diagnosed with ASD at 36 months	6–24 months, assessment every 6 months	Developmental study, no control group	ADOS, Bayley Scales of Infant Development, 2nd ed. or MSEL, CDI-Words and Gestures, Infant Temperament Scale or Toddler Behavior Assessment Questionnaire	Two groups were identified: 1st subgroup (*n =* 6) showed a decrease in IQ between 12 and 24 or 36 months; 2nd subgroup (*n =* 3) continued to obtain average IQs. Signs of autism emerged and/or were more striking earlier in the 1st group (*n =* 6). In all children early impairment in social-communicative development coexisted with atypical sensory and/or motor behaviors and temperamental profile marked by irritability/distress and dysregulated state
Cassel *et al.* [91]	*J. Autism Dev. Disord.*, 2007	Social and emotional communication	12 infant siblings of children with autism	6–18 months	19 age-matched TD control children	Face-to-face/still-face (FFSF), Early Social Communication Scale (ESCS)	Siblings smiled for a lower proportion of the FFSF than TD and lacked emotional continuity between episodes. Siblings engaged in lower rates of initiating joint attention at 15 months, lower rates of higher-level behavioral requests at 12 months, and responded to fewer joint attention bids at 18 months. Infant siblings experience subtle, inconsistent, but multi-faceted deficits in emotional expression and referential communication
Iverson *et al.* [92]	*J. Autism Dev. Disord.*, 2007	Vocal-motor development	21 infant siblings of children with autism	5–18 months	18 TD control children, maternal and parental age and levels of parental education comparable in sample and control group	Videotaping: naturalistic observation, semi-structured play, play in a Johnny Jump-Up, face-to-face interaction, and play with toys; MacArthur–Bates Communicative Development Inventory; Pervasive Developmental Disorder Screening Test-II	Infant siblings were delayed in the onset of early developmental milestones and spent significantly less time in a greater number of postures, suggestive of relative postural instability. Infant siblings demonstrated attenuated patterns of change in rhythmic arm activity around the time of reduplicated babble onset; and were highly likely to exhibit delayed language development at 18 months
Loh *et al.* [108]	*J. Autism Dev. Disord.*, 2007	Stereotyped motor behaviors	8 infant siblings of children with autism later diagnosed with ASD, 9 infant siblings of children with autism not diagnosed with ASD	12 and 18 months	15 TD control children, same geographic area as the sibling sample and age-matched to the high-risk infants	Measurement of Repetitive Motor Behaviors, videotaping	At 12 and 18 months the ASD group “arm waved” more frequently and at 18 months, one posture (“hands to ears”) was more frequently observed in the ASD and non-diagnosed group compared to the TD
Merin *et al.* [93]	*J. Autism Dev. Disord.*, 2007	Visual fixation patterns during reciprocal social interaction	31 infant siblings of children with autism	6 months	24 Comparison infants with no autism family history, age and gender controlled	Modified Still Face paradigm, Eye tracking	Eleven infants demonstrated diminished gaze to the mother’s eyes relative to her mouth during the Still Face episode; 10 of them had an older sibling with ASD
Stone *et al.* [103]	*Arch. Pediatr. Adolesc. Med.*, 2007	Communicative and cognitive development	64 siblings of children with ASD (SIBS-A)	12–23 months	42 control children with no autism family history, no matching criteria except for age range	MSEL, CARS, Screening Tool for Autism in Two-Year-Olds (STAT), MacArthur Communicative Development Inventories, Detection of Autism by Infant Sociability Interview	Younger siblings of children with ASD demonstrated poorer performance in nonverbal problem solving, directing attention, understanding words, understanding phrases, gesture use, and social-communicative interactions with parents, and had increased autism symptoms relative to control siblings
Sullivan *et al.* [98]	*J. Autism Dev. Disord.*, 2007	Response to joint attention	51 infant siblings of children with autism; Outcome groups at age 3 years: 16 ASD, 8 broader autism phenotype, and 27 non-broader autism phenotype	14 and 24 months	Developmental study, no control group	Adaptation of a task described by Butterworth and Jarrett (1991) to assess response to joint attention; the Communication and Symbolic Behavior Scales Developmental Profile, MSEL, ADOS	Lower response to joint attention was observed for the ASD group at 24 months. Response to joint attention performance at 14 months predicted ASD outcome. The ASD group made minimal improvement in response to joint attention between 14 and 24 months
Toth *et al.* [94]	*J. Autism Dev. Disord.*, 2007	Social, imitation, play and language abilities	42 non-autistic siblings of children with autism 20 toddlers with no family history of autism	18–27 months	20 toddlers with no family history of autism, controlled for age and ethnic group	MSEL, The Vineland Social-Emotional Early Childhood Scales, The Communication and Symbolic Behavior Scale-Developmental Profile, Imitation battery developed by Meltzoff, The Play Assessment Scale, The Early Development Interview	Siblings scored poorer in Receptive language scale (MSEL), Daily living skills, Motor and Composite (Vineland)
Yirmiya *et al.* [89]	*J. Autism Dev. Disord.*, 2007	Cognitive and language profile —a prospective study	30 siblings of children with autism (SIBS-A)	24–36 months	30 siblings of typically developing children (SIBS-TD); matched on the basis of chronological age, gender, birth order, scores on mental and psychomotor indices, temperamental characteristics and number of children in the family	BSID-II, RDLS, CHAT, K-ABC, CELF-P, The Social and Communication Questionnaire (SCQ)	At 24 months: more SIBS-A demonstrated language scores one or two standard deviations below the mean compared to SIBS-TD. At 36 months: more SIBS-A displayed receptive and expressive difficulties compared to SIBS-TD. Six SIBS-A (including one diagnosed with autism) revealed language scores more than two standard deviations below the mean at both ages, a pattern not seen in the SIBS-TD
Garon *et al.* [116]	*J. Abnorm. Child Psychol.*, 2009	Temperamental traits	138 HR infants with an older sibling with autistic spectrum disorder	6–36 months	73 low risk infants with no family history of ASD, no matching criteria listed except for age range	Toddler Behavior Assessment Questionnaire-Revised, MSEL, ADOS, ADI-R	HR children, who were diagnosed with ASD at 36 months, had temperament profile marked by lower positive affect, higher negative affect, and difficulty controlling attention and behavior (labelled as Effortful Emotion Regulation). This temperamental profile distinguished also HR children without ASD diagnosis at 36 months from LR children
Yoder *et al.* [67]	*J. Autism Dev. Disord.*, 2009	Social impairment and ASD diagnosis	43 siblings of children with autism (SIBS-ASD)	15–34 months	24 SIBS-TD, matched on the basis of child’s age and maternal education	MSEL, STAT, Responding to Joint Attention (RJA), Social Behavior Checklist (SBC), ADOS, ADI-R	Initial level of responding to joint attention and growth rate of weighted triadic communication predicted the degree of social impairment at the final measurement period of SIBS-ASD. Both predictors were associated with later ASD diagnosis, contrary to unweighted triadic communication, age of entry into the study, and initial language level, which did not predict later social impairment
Christensen *et al.* [117]	*J. Autism Dev. Disord.*, 2010	Play behaviors	17 ASD siblings later diagnosed with ASD, infant siblings of children with autism with and without other delays (Other Delays and No Delays siblings; *n =* 12 and *n =* 19, respectively)	18 months	19 TD children, no matching criteria listed except for age range	Free-play task: functional, symbolic, and repeated play actions	ASD siblings showed fewer functional and more non-functional repeated play behaviors than TD children. Other Delays siblings showed more non-functional repeated play than TD controls. Group differences disappeared with the inclusion of verbal mental age
Holmboe *et al.* [109]	*Infant Behav. Dev.*, 2010	Executive functions, attention and inhibition, frontal cortex functioning	31 SIBS-ASD	9–10 months	33 typically developing children with no family history of autism, no matching criteria listed except for age range	Freeze-Frame task	SIBS-ASD had difficulty disengaging attention and showed less selective inhibition than controls (less difference between interesting and boring trials); however, they demonstrated selective inhibitory learning (tendency to show a larger decrease in looks to the distractors in the interesting trials than in the boring trials, whereas controls showed a similar decrease in the two trial type)
Haltigan *et al.* [113]	*J. Autism Dev. Disord.*, 2011	Attachment security	51 infant siblings of older children with ASD (SIBS-ASD)	15 months	34 typically developing children with no family history of autism (SIBS-COMP); no matching criteria listed except for age range	Strange Situation Procedure (SSP)	SIBS-ASD are not less likely to form secure affectional bonds with their caregivers than SIBS-COMP. Larger rate of B1–B2 secure subclassifications in SIBS-ASD than controls (B1–B2 infants are less distressed during separation and are more reserved after reunion with caregiver)
Ozonoff *et al.* [31]	*Pediatrics*, 2011	Recurrence risk for ASD	664 infants with an older sibling with ASD	18–36 months	No control group, developmental study	ADOS, MSEL	18.7% of the infants developed ASD. Infant gender (threefold increase in risk for male subjects) and the presence of 1 affected older sibling (twofold increase in risk) were significant predictors of ASD outcome
Paul *et al.* [118]	*J. Child Psychol. Psychiatry*, 2011	Vocal production	At 6 months: 28 high-risk (HR) infants; at 9 months: 37 HR infants; at 12 months: 38 HR infants; at 24 months: 24 HR infants	6–24 months	At 6 months: 20 low-risk (LR) infants; at 9 months: 29 LR infants; at 12 months: 31 LR infants; at 24 months: 21 LR infants; no matching criteria listed except for age range	ADOS, MSEL, Vocalization Sample Collection	Differences were seen between risk groups for certain vocal behaviors. Differences in vocal production in the first year of life were associated with outcomes in terms of autistic symptomatology in the second year
Rozga *et al.* [97]	*J. Autism Dev. Disord.*, 2011	Mother–infant interaction and nonverbal communication, social gaze, affect, and joint attention behaviors	17 infant siblings of older children with ASD, later diagnosed with autism; 84 infant siblings of older children with ASD without ASD diagnosis (NoASD-sib)	6–36 months	66 TD children, no matching criteria listed except for age range	Free Play Mother–Infant Interaction, Still Face Procedure, ESCS	The ASD group did not differ from the other two groups at six months in the frequency of gaze, smiles and vocalizations directed toward the caregiver, nor in their sensitivity to her withdrawal from interaction. By 12 months, infants in the ASD group exhibited lower rates of joint attention and requesting behaviors. NoASD-sibs did not differ from comparison infants on any variables of interest at 6 and 12 months
Bedford *et al.* [111]	*J. Autism Dev. Disord.*, 2012	Social gaze, communication and attentional engagement	54 HR infants	7 and 13 months	50 LR infants, no matching criteria listed except for age range	Eye-tracking	No group difference between high-risk and low-risk infants in gaze-following behavior at either age. At-risk infants with later emerging socio- communication difficulties (ASD and atypical development) allocated less attention to the congruent object compared to typically developing high-risk siblings and low-risk controls
Cornew *et al.* [119]	*J. Autism Dev. Disord.*, 2012	Social referencing	38 HR infants	17.7–20.6 months	44 LR infants; LR and HR groups controlled for equality of mean age and infants’ maturity at birth	Social referencing procedure	Compared to both typically developing infants and high-risk infants without ASD, infants later diagnosed with ASD engaged in slower information seeking. High-risk infants, both those who were and those who were not later diagnosed with ASD, exhibited impairments in regulating their behavior based on the adults’ emotional signals
Dundas *et al.* [115]	*J. Autism Dev. Disord.*, 2012	Face processing	43 HR infants	6 and 11 months	31 LR infants; gender, ethnicity, and age of infants in LR and HR groups described	Eye-tracking	Low-risk infants demonstrated a preference for looking at the left side of the face, which emerged by 11 months of age. High-risk infants did not demonstrate a left visual field bias at either age
Hutman *et al.* [95]	*J. Autism Dev. Disord.*, 2012	Social interactions, selective visual attention	81 HR infants; Outcome groups: 15 ASD; 12 Other Concerns; 59 High-Risk Typical; and 43 Low-Risk Typical	12 months	48 LR infants, no matching criteria listed except for age range	Examiner–Child Interaction, play, and distress condition	No difference in proportion of attention to social stimuli or attention shifting during the play condition between groups. Infants later diagnosed with ASD tended to continue looking at a toy during the distress condition despite the salience of social information. Emotion recognition is intact in infants who later develop autism, but the emotional value of the information appears to be less salient
Macari *et al.* [101]	*J. Autism Dev. Disord.*, 2012	Risk for ASD	53 HR infants	12, 18 and 24 months	31 LR infants; no matching criteria listed except for age range	MSEL, ADOS-Toddler	About 2/3 of infants at high risk for ASD experience some kind of developmental difficulties in the second year of life
Curtin &Vouloumanos [120]	*J. Autism Dev. Disord.*, 2013	Speech preference	31 HR infants	12–18 months	31 LR infants; no matching criteria listed except for age range	Speech/Non-Speech task, MSEL, MacArthur–Bates Communicative Development Inventories	Only low-risk infants listened significantly longer to speech than to non-speech at 12 months. In both groups, relative preference for speech correlated positively with general cognitive ability at 12 months. However, in high-risk infants only, preference for speech was associated with autistic-like behavior at 18 months, while in low-risk infants, preference for speech correlated with language abilities
Damiano *et al.* [110]	*J. Autism Dev. Disord.*, 2013	Repetitive and stereotyped movements	20 HR infants (SIBS-ASD)	15–24 months	20 typically developing siblings (SIBS-TD), differences in maternal educational between HR and LR groups were noted	STAT, Repetitive and Stereotyped Movement Scales (RSMS)	SIBS-ASD displayed higher rates of repetitive and stereotyped movements (RSM) relative to SIBS-TD; SIBS-ASD as a group demonstrated a significantly higher inventory of RSMs than controls, but this difference was no longer significant after excluding subgroup of ASD diagnosed SIBS-ASD. Different patterns for Object vs. Body RSM inventory for the high-risk groups with different diagnostic outcomes (Sibs-ASD/+ *vs*. Sibs-ASD/−)
Georgiades *et al.* [100]	*JAMA Psychiatry*, 2013	Autistic-like traits	170 HR infants	12 months	90 LR control subjects with no family history of ASD; no matching criteria listed except for age range	The Autism Observation Scale for Infants	Cluster 1 (*n =* 37), having significantly higher levels of autistic-like traits, consisted of 33 children from the siblings and only four from the control subjects. At the age of three, children from cluster 1 had more social-communication impairments, lower cognitive abilities, and more internalizing problems. Nineteen percent of HR siblings who did not meet ASD diagnostic criteria at the age of three showed autistic-like traits resembling a BAP by 12 months
Messinger *et al*. [88]	*J. Am. Acad. Child Adolesc. Psychiatry*, 2013	ASD risk, autistic traits	507 HR siblings	8–36 months	324 LR control subjects, no matching criteria listed except for age range	ADOS calibrated severity scores, and Mullen Verbal and Non-Verbal Developmental Quotients (DQ)	At three years, HR siblings without an ASD outcome exhibited higher mean ADOS severity scores and lower verbal and non-verbal DQs than LR controls. HR siblings were over-represented (21% HR vs. 7% LR) in latent classes characterized by elevated ADOS severity and/or low to low-average DQs. The remaining HR siblings without ASD outcomes (79%) belonged to classes in which they were not differentially represented with respect to LR siblings
Wan *et al*. [114]	*J. Child Psychol. Psychiatry*, 2013	Quality of interaction	At 6–10 months: 45 HR infants; at 12–15 months: 43 HR siblings	6–10 and 12–15 months	At 6–10 months: 47 LR siblings; at 12–15 months: 48 LR siblings; no matching criteria listed except for age range	Six-min videotaped episodes of parent-infant free play; Manchester Assessment of Caregiver-Infant Interaction (MACI)	At six months, infant liveliness was lower in the at-risk groups; at 12 months, infant attentiveness to parent and positive affect were lower in the at-risk group later diagnosed with ASD. Dyadic mutuality, infant positive affect and infant attentiveness to parent at 12 months predicted three-year ASD outcome, whereas infant ASD-related behavioral atypicality did not
Del Rosario *et al.* [121]	*J. Autism Dev. Disord.*, 2014	Temperament trajectories	16 HR infants who were later diagnosed with ASD (SIBS-ASD), 27 HR infants who demonstrated typical patterns of development (SIBS-HR-TD)	6–36 months	No control group	Carey Temperament Scales completed by parents, MSEL	Temperament trajectories of children with ASD reflected increases over time in activity level, and decreasing adaptability and approach behaviors relative to high-risk typically developing (HR-TD) children
Esposito *et al.* [112]	*J. Autism Dev. Disord.*, 2014	Expression of distress during the separation phase	13 HR infants	15 months	14 LR infants; LR and HR groups were controlled for the age of infants and the age of older siblings	Cry samples derived from vocal recordings	HR toddlers, compared to those with LR, produced cries that were shorter and had a higher fundamental frequency (F0). Three HR toddlers later classified with an ASD at 36 months produced cries that had among the highest F0 and shortest durations
Gangi *et al.* [106]	*J. Autism Dev. Disord.*, 2014	Joint attention initiation, social communication	56 HR siblings	8–12 months	26 LR siblings; LR and HR groups were controlled for age and ethnicity	Initiating joint attention (IJA) smiling patterns (*i.e.*, anticipatory smiling, reactive smiling, and no smiling) assessed with Early Social Communication Scales	High-risk siblings produced less anticipatory smiling than low-risk siblings, suggesting early differences in communicating pre-existing positive affect. Among high-risk siblings, only IJA without smiling was associated with later ASD severity scores
Gliga *et al.* [122]	*Dev. Psychol*., 2014	Spontaneous belief attribution, mental state understanding for action prediction	47 siblings of children with ASD	36 months	39 typically developing children; no matching criteria listed except for age range	Eye-tracking	In tasks demanding mental state understanding for action prediction, at-risk siblings performed at chance (contrary to control children, who performed above the chance), independently of their later clinical outcome (ASD, broader autism phenotype, or typically developing). Performance was not related to children’s verbal or general IQ, nor was it explained by children “missing out” on crucial information, as shown by an analysis of visual scanning during the task
Hudry *et al.* [104]	*J. Autism Dev. Disord.*, 2014	Early language profiles, communication	54 HR infants	7–38 months	50 LR controls, no matching criteria listed except for the age range	MCDI: Words and Gestures (WG) and Words and Sentences (WS), VABS—2nd edition and MSEL	Reduced receptive vocabulary advantage was observed in HR infants by 14 months, but was maintained to 24 months only in children with ASD outcome, while typically-developing HR infants regained a more normative profile
Nichols *et al.* [107]	*J. Autism Dev. Disord.*, 2014	Social communication, social smiling	15 SIBS-ASD/AS (siblings of children with ASD, who demonstrated later ASD symptomatology), 27 SIBS-ASD/NS (siblings of children with ASD , who did not demonstrate ASD symptoms)	15 months	25 siblings of children with no family history of ASD, SIBS-TD; no matching criteria listed except for the age range	MSEL, STAT, ADOS	Both SIBS-ASD subgroups demonstrated lower levels of social smiling than SIBS-TD. Only the SIBS-ASD/AS demonstrated less eye contact and non-social smiling than SIBS-TD
Patten *et al.* [123]	*J. Autism Dev. Disord.*, 2014	Vocal patterns, vocalization frequency	37 HR infants (23 obtained the ASD diagnosis later)	9–12 and 15–18 months	14 typically developing infants with no autism family history (LR); HR and LR groups did not differ in terms of age, gender or SES	Video records	Infants later diagnosed with ASD produced low rates of canonical babbling and low volubility by comparison with the typically developing infants

Some authors have posited alternative temperament development trajectories in HR infants, characteristic for early ASD symptoms or broader autism phenotype condition. HR children who were diagnosed with ASD at 36 months had a temperament profile marked by lower positive affect, higher negative affect, and difficulty controlling attention and behavior. This temperamental profile also distinguished HR children without ASD diagnosis at 36 months from LR children [116]. Rosario *et al.* [121] compared HR infants who were or were not later diagnosed with ASD and discovered that the temperament trajectories of children with ASD reflected increases over time in activity levels and decreasing adaptability and approach behaviors relative to high-risk typically developing (TD) children.

It is also predicted that HR infants will exhibit problems with executive functions, especially attention and inhibition deficits, which are considered to be associated with frontal cortex functioning impairments [109]. HR infants had difficulty disengaging attention and showed less selective inhibition than controls (less difference between interesting and boring trials); however, they demonstrated a larger decrease in looks to the distractors in the interesting trials than in the boring trials, whereas controls showed a similar decrease in the two trial types.

Studies investigating vocalization patterns in HR infants suggest that infants later diagnosed with ASD produce low rates of canonical babbling and low volubility in comparison with typically developing infants [123]. Iverson *et al.* [92] also found impaired vocal-motor developmental behaviors in HR infants. Infant siblings demonstrated attenuated patterns of change in rhythmic arm activity around the time of reduplicated babble onset, and were highly likely to exhibit delayed language development at 18 months.

It should be stressed that the analyzed studies have many limitations. As previously mentioned, some authors include in the analysis high-risk infant siblings who later developed ASD, which can lead to overestimation of the differences between high-risk and low-risk children, *i.e.*, [95,97,111]. Furthermore, some studies do not report any clear matching criteria of the control group participants during the recruitment procedure—there is only a description provided of recruited participants in terms of different parameters (such as gender, age, ethnicity, *etc.*), *i.e.*, [110,115,119]. It should be noted that these features often vary across studies, which can raise questions about the comparability and recurrence of obtained results with other research projects. There are also studies that do not have any control group at all, because of the aim of the study, *i.e.*, comparing HR siblings who later developed ASD or did not [107,121]. These research projects provide information about differences within the HR group, but lack data from comparisons of these HR infants with typically developing children that do not have an ASD family background. There are also discrepancies between infants’ age ranges in different studies. Some papers focus on describing the features of children at exactly the same age (*i.e.*, 6, 12, or 24 months [102], or other time points [30,90,96]), while others collect data covering a fixed age range (18–27 months [94], below 3 years old [105], 12–23 months [103]). These distinct approaches produce different types of data and therefore variable quality of comparisons, which should be considered during an analysis of studies.

In summary, there are many studies suggesting the existence of a broad range of impairments in infant siblings of children with ASD. Some of the differences are probably characteristic for later ASD diagnosis, but it should be noted that infant siblings are also at a high risk of developing broader autism phenotype-like traits.

## 4. Summary and Conclusions

The review of studies on BAP traits in siblings of individuals with ASD shows that the issue has been extensively explored. This goes hand in hand with investigation of hereditary mechanisms involved in the etiology of autism spectrum disorders. Research on BAP provides important information about the varied expressions of autism-specific traits. Integration of the results of these studies presents a challenge due to differences in methods, control groups, age of participants, and other aspects of research protocols. Since the relationships between autistic traits and other individual characteristics in a general population, including first-degree relatives of individuals with ASD, have not yet been fully elaborated, this paper focuses on the phenotypic characteristics of individuals observed mainly in psychological studies.

A number of investigators reported cognitive deficits in siblings of children with ASD, which can be a part of ASD phenotype(s). These include emotion recognition tasks [52], lower levels of efficiency in planning, attention shifting, and verbal fluency [44,48]. Some studies indicated differences in social skills development [6,14,28,69]. Difficulties for siblings of children with ASD were also found with respect to communication and language. There are reports of histories of language delay and pragmatic language deficits in this group compared with the siblings of children with Down syndrome and typically developing children, *i.e.*, [28,60,61,94,102].

Researchers have posited various BAP traits in siblings of individuals with autism as important components of the neurocognitive endophenotype for autism. For instance, Dalton *et al.* [55] suggested that social and emotional processing along with underlying neural circuitry constitute an important element of the endophenotype. Fiorentini and colleagues [63] point to face-coding mechanisms, emphasizing their role in the impairment of adaptive mechanisms, while Holt and colleagues [22] highlight the role of mentalizing deficits and atypical social cognition. The inclusion of language deficits in BAP remains controversial. Based on their findings from research on children aged 4–9 years, Levy and Bar-Yuda [61] question whether these deficits should indeed be included in BAP, while Gamliel *et al.* [56,57] consider them to be the key component in the difficulties encountered by preschool and early school individuals with ASD.

It should also be noted that some researchers found no differences between siblings of people with ASD and comparison groups with respect to BAP characteristics [40,45]. Importantly, in a number of studies subgroups demonstrating developmental deficits were distinguished from the group of healthy siblings of people with autism [39,65]. The clear distinction between ASD symptoms and BAP traits is difficult in studies involving siblings of individuals with ASD. This is especially true in studies on infant siblings, in which the risk of ASD rather than the BAP characteristics is the main concern. Prospective studies in this group of children should be continued, in order to track the developmental trajectories in these children at subsequent stages of their development.

It should be stressed that in the light of BAP research, the majority of brothers and sisters of individuals with ASD develop typically, without displaying autistic traits to a greater extent than the relevant control groups. However, traits in the siblings group are often widely dispersed, suggesting that there is much variation among these children in the course of developmental processes and their outcomes.

Some data suggest that genetic susceptibility to autism may differ among families. It is likely to be higher in families with two or more children diagnosed with ASD. Other siblings in these families demonstrate more pronounced BAP traits [65,124]. Research on these families could provide valuable insights on genetic involvement in the development of autistic traits.

Longitudinal studies are especially useful, as they enable researchers to trace developmental dynamics of ASD siblings. Despite achieving the status of the gold standard in research on infants, more longitudinal studies on children over 36 months of age are needed.

The information available at present is insufficient to formulate final conclusions regarding BAP characteristics in siblings of people with ASD. There is no doubt, however, that current research is bringing us closer to an understanding of the genetic factors involved in the etiology of this group of disorders. The studies are also of fundamental importance due to the rising numbers of ASD diagnoses and the presence of autistic characteristics in the general population.
